# Aurora A kinase inhibition enhances oncolytic herpes virotherapy through cytotoxic synergy and innate cellular immune modulation

**DOI:** 10.18632/oncotarget.14885

**Published:** 2017-02-02

**Authors:** Mark A. Currier, Les Sprague, Tilat A. Rizvi, Brooke Nartker, Chun-Yu Chen, Pin-Yi Wang, Brian J. Hutzen, Meghan R. Franczek, Ami V. Patel, Katherine E. Chaney, Keri A. Streby, Jeffrey A. Ecsedy, Joe Conner, Nancy Ratner, Timothy P. Cripe

**Affiliations:** ^1^ Center for Childhood Cancer and Blood Diseases, Nationwide Childrens Hospital, The Ohio State University, Columbus, Ohio, USA; ^2^ Division of Experimental Hematology and Cancer Biology, Cincinnati Childrens Hospital Medical Center; Cincinnati, Ohio, USA; ^3^ Division of Hematology/Oncology/Blood and Marrow Transplantation, Nationwide Childrens Hospital, The Ohio State University, Columbus, Ohio, USA; ^4^ Takeda Pharmaceuticals International Co, Cambridge, MA, USA; ^5^ Virttu Biologics, Ltd, Biocity, Scotland, Newhouse, United Kingdom

**Keywords:** oHSV, Aurora A kinase, neuroblastoma, MPNST

## Abstract

Malignant peripheral nerve sheath tumor (MPNST) and neuroblastoma models respond to the investigational small molecule Aurora A kinase inhibitor, alisertib. We previously reported that MPNST and neuroblastomas are also susceptible to oncolytic herpes virus (oHSV) therapy. Herein, we show that combination of alisertib and HSV1716, a virus derived from HSV-1 and attenuated by deletion of RL1, exhibits significantly increased antitumor efficacy compared to either monotherapy. Alisertib and HSV1716 reduced tumor growth and increased survival in two xenograft models of MPNST and neuroblastoma. We found the enhanced antitumor effect was due to multiple mechanisms that likely each contribute to the combination effect. First, oncolytic herpes virus increased the sensitivity of uninfected cells to alisertib cytotoxicity, a process we term virus-induced therapeutic adjuvant (VITA). Second, alisertib increased peak virus production and slowed virus clearance from tumors, both likely a consequence of it preventing virus-mediated increase of intratumoral NK cells. We also found that alisertib inhibited virus-induced accumulation of intratumoral myeloid derived suppressor cells, which normally are protumorigenic. Our data suggest that clinical trials of the combination of oHSV and alisertib are warranted in patients with neuroblastoma or MPNST.

## BACKGROUND

Aurora A Kinase is a serine/threonine kinase that serves many functions in cell division, being both directly and indirectly involved with centrosome formation, spindle assembly, and cytokinesis [1–3]. Overexpression of Aurora A Kinase has been observed in multiple malignancies including bladder, breast, colon, pancreatic, malignant peripheral nerve sheath tumor (MPNST), and neuroblastoma [1, 3, 4]. Aurora A Kinase acts as an oncoprotein when transfected to target cells [5], and interestingly, Aurora A Kinase up-regulation in cancer correlates with resistance to apoptosis [6]. Due to its involvement in mitosis and association with cancer, small molecule Aurora A Kinase inhibitors, including alisertib (MLN8237), are currently under investigation as therapeutics in numerous cancer types. Multiple reports underscore the anti-tumor efficacy of alisertib in tumor models where it causes cell cycle arrest and apoptosis in cancer cells [3].

HSV1716 is an oncolytic herpes simplex virus (oHSV) derived from wild type HSV-1 in which the RL1 gene, encoding the protein ICP34.5 known as the “neurovirulence factor,” was deleted from the viral genome [7]. In wild type HSV-1, the ICP34.5 gene product counteracts host protein kinase R (PKR) activated by the anti-viral interferon response to infection [7–9]. Without ICP34.5 expression, interferon responses in normal tissues result in PKR activation and phosphorylation of the ribosomal eIF2α subunit, leading to the arrest of both protein synthesis and virus production. Many cancer types exhibit dysfunctional interferon signaling and/or activation of the MAP kinase pathway, which inhibits PKR [10], rendering cells particularly susceptible to ICP34.5-null HSV-1. Therefore, ICP34.5 deletion allows HSV1716 to selectively infect and replicate in tumor cells, while sparing healthy tissues.

Previous studies from our group and others show that a wide variety of tumor types are susceptible to infection with oHSV, including the neuralcrest-derived peripheral nerve tumors, neuroblastoma and MPNST [11–14]. Infected cells typically undergo virus-mediated cytolysis, produce immunomodulatory cytokines, and release danger signals that can lead to cell death in infected and surrounding cells [15]. While the safety, tolerability, and efficacy of HSV1716 have been demonstrated in preclinical models and several human clinical trials [16–19], it is unlikely to induce cancer cures as a monotherapy. Therefore, it is of interest to utilize pharmacologic agents in combination with HSV1716 to find increasingly effective combination therapies.

Here we report that the combination of alisertib and HSV1716 is synergistic. As a mechanism for the enhanced cell killing, we found that oHSV infected cells exert a paracrine effect on uninfected cells that increases their sensitivity to alisertib. We also found that alisertib enhances virus production in tumors and slows immune-mediated virus clearance by reducing innate immune cell infiltrates. Together, our data suggest that the superior outcome of HSV1716 plus alisertib in xenograft models of MPNST and neuroblastoma can be attributed to multiple diverse mechanisms.

## RESULTS

### Alisertib and HSV1716 are synergistic *in vivo*

**Figure 1:**
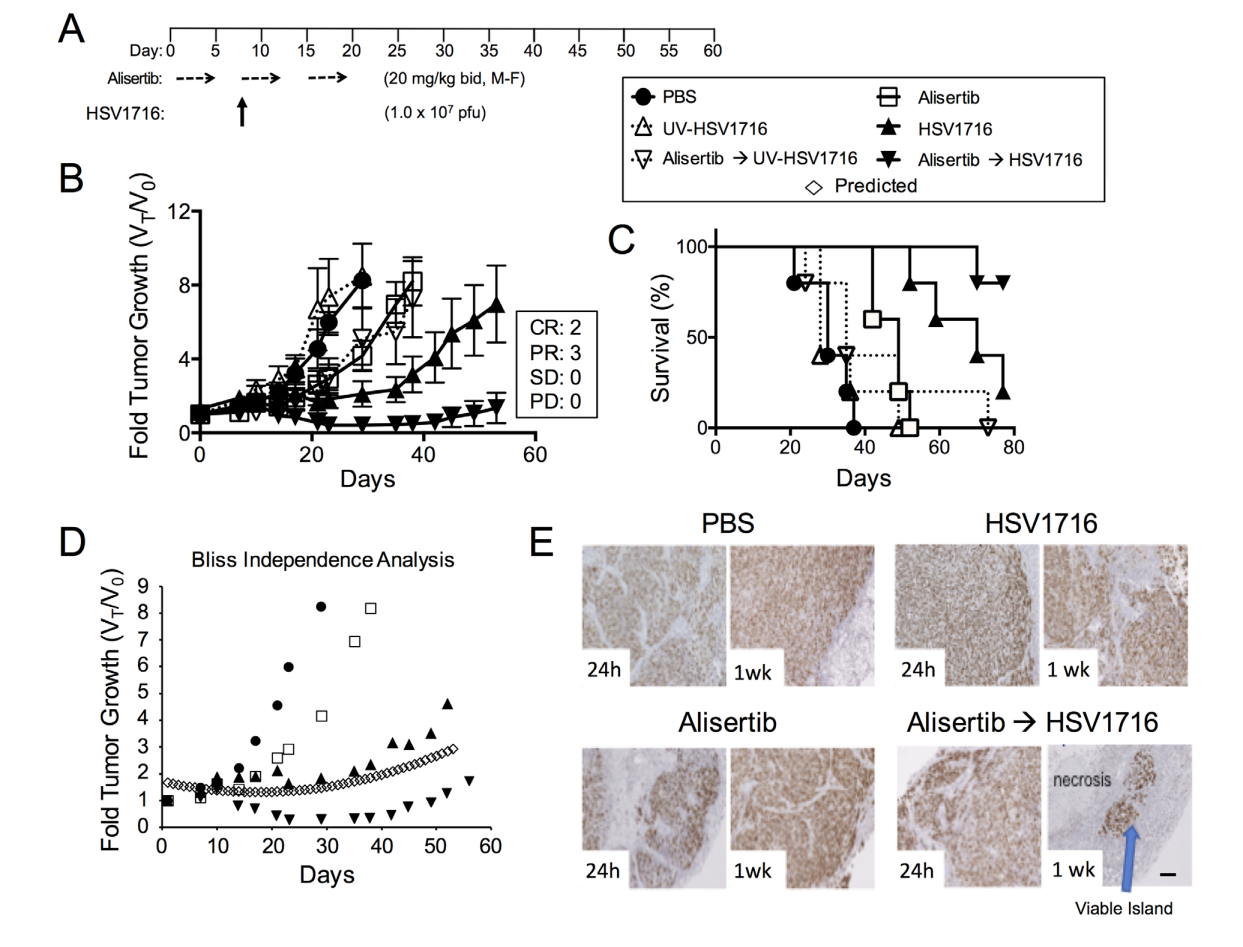
Alisertib and HSV1716 are synergistic in MPNST xenografts **A**. Mice bearing subcutaneous S462TY xenograft tumors were given alisertib (20 mg/kg) or vehicle by oral gavage (twice daily 5 days per week M=Monday, F=Friday) for 19 days in addition to a single intratumoral injection of HSV1716 (1.0 × 10^7^ pfu), UV-HSV1716 or saline (PBS) at day 7 (*n* = 5). **B**. Tumors were measured twice per week and growth plotted as fold tumor growth. Clinical responses were categorized for each tumor in the combination group as shown complete response (CR), partial response (PR), stable disease (SD) or progressive disease (PD). **C**. Mice were sacrificed when the tumor volume reached 1500mm^3^ (*n* = 5, p value for survival determined by log-rank). **D**. Graphical comparison of actual versus predicted (open diamonds) growth of tumors treated with combination as determined by the Bliss Independence Analysis. **E**. Mouse tumors were assessed by Ki67 staining at 24 hours and 1 week post infection. Areas of proliferating tumor cells were identified by Ki67 staining. Scale bar: 100 μM.

We used models of malignant peripheral nerve sheath tumors (MPNST) and neuroblastoma because previous studies by us and others show susceptibility to alisertib and oHSV as single agents [3, 4, 11, 12, 20, 21]. Compared with vehicle controls, treatment of S462TY MPNST tumor-bearing animals with alisertib at 20 mg/kg twice daily slowed tumor growth resulting in increased overall survival (Figure 1, *p* = 0.0072). Mice treated with HSV1716 as a single agent showed even more growth inhibition and enhanced overall survival (*p* = 0.0021). The combination of alisertib plus HSV1716 was the most potent. We observed objective responses in all combination treated animals, including 3 partial responses (PR) and 2 complete responses (CR) and 100% survival through day 80 (60 days after treatment ended). The effect was greater than additive as determined by the Bliss Independence Analysis of the tumor growth curves (Figure 1D). On histology, tumors showed massive necrotic areas with only small islands of viable tumor (stained with Ki67) only in the combination group (Figure 1E). All groups tolerated the dosing regimen well based on body weight change (data not shown). By using a virus inactivated by ultraviolet irradiation (UV-HSV1716), we confirmed that active virus replication is required for either single or combination efficacy (Figure 1B and 1C).

**Figure 2:**
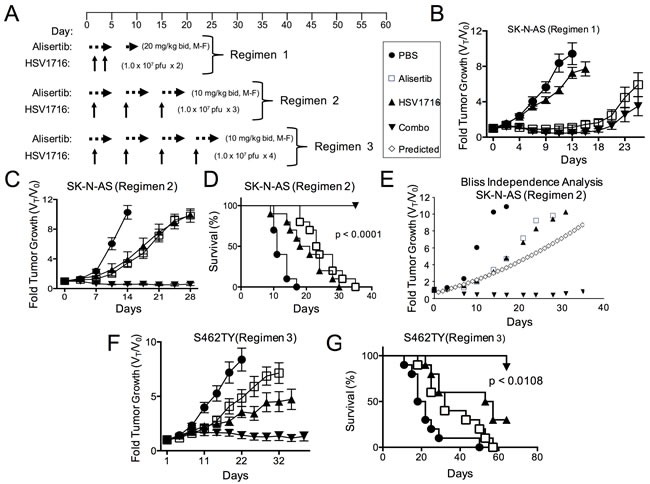
Alisertib and HSV1716 are synergistic in neuroblastoma xenografts **A**. A schematic illustrating the multiple dosing regimens for mice bearing subcutaneous SK-N-AS or S462TY xenograft tumors. **B**. Mice bearing subcutaneous SK-N-AS xenograft tumors were first given alisertib (20 mg/kg) or vehicle by oral gavage (twice daily 5 days per week, M-F) for 9 days in addition to an intratumoral injection of HSV1716 (1 × 10^7^ pfu) or PBS on days 2 and 4. **C**. Mice bearing subcutaneous SK-N-AS xenograft tumors were given alisertib (10 mg/kg) or vehicle by oral gavage (twice daily 5 days per week) for 19 days in addition to intratumoral injections of HSV1716 (1 × 10^7^ pfu) or PBS on days 5, 12 and 19. **D**. Survival curves of mice in panel C; mice were sacrificed when the tumor volume reached 1500mm^3^ (*n* = 10, *p*-value for survival determined by log-rank). **E**. Graphical comparison of actual versus predicted growth of tumors treated with combination as determined by the Bliss Independence Analysis. **F**. Mice bearing subcutaneous S462TY xenograft tumors were administered alisertib (10 mg/kg) or vehicle by oral gavage (twice daily 5 days per week) for 26 days in addition to an intratumoral injection of HSV1716 (1.0 × 10^7^ pfu) or PBS on days 5, 12, 19 and 26. **G**. Survival curves of mice in panel F; mice were sacrificed when the tumor volume reached 1500mm^3^ (PBS control (*n* = 9), alisertib (*n* = 10), HSV1716 (*n* = 10) and alisertib plus HSV1716 (combo, *n* = 8)); p value for survival determined by log-rank)

In our prior study of virus alone, some xenograft models of neuroblastoma were highly sensitive to virus, but others were less sensitive, with the most resistant being SK-N-AS [12]. Because we found SK-N-AS significantly less susceptible to virus transduction than S462TY (16% positive versus 66% at MOI = 1 following exposure to HSV1716-GFP, respectively), we modified our treatment regimen to include more virus doses in the SK-N-AS model (Figure 2A). Interestingly, alisertib at 20 mg/kg alone was nearly as effective as alisertib plus HSV1716 in inhibiting SK-N-AS tumor growth with 7 of 8 responses (either CR, PR, or stable disease (SD)) versus 10 of 10 responses, respectively (Figure 2B). Thus in order to ascertain any combination activity, we reduced alisertib to 10 mg/kg once daily, which better mimics feasible clinical exposures, combined with repeated weekly injections of HSV1716 for a total of 3 injections. Using this regimen, either alisertib or HSV1716 alone slowed tumor growth. The combination inhibited tumor growth, an effect that lasted well beyond the end of treatment and enabled prolonged animal survival (Figure 2C and 2D). Again, the effect was greater than additive as determined by the Bliss Independence Analysis of the tumor growth curves (Figure 2E). We also confirmed this reduced dose of alisertib with virus retained combination efficacy in S462TY tumors (Figure 2F and 2G).

### Alisertib and HSV1716 show synergistic cytotoxicity

**Figure 3:**
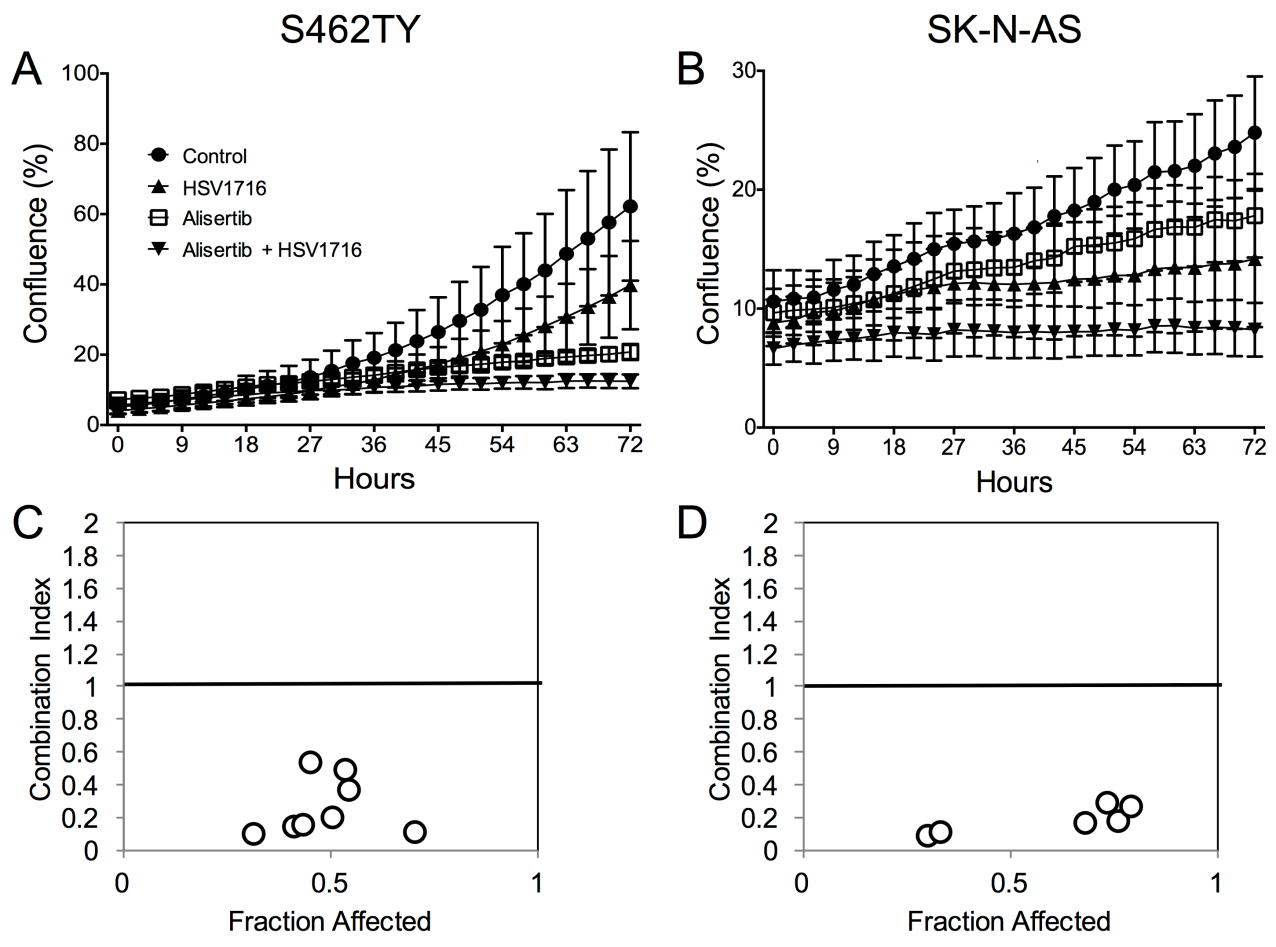
Alisertib and HSV1716 are synergistic ***in vitro***. **A**. S462TY and **B**. SK-N-AS cells were treated with HSV1716-GFP (MOI = 0.5) alone, alisertib (50 nM) or in combination. The Incucyte ZOOM live cell imager was used to monitor cell confluence over time. **C**. and **D**. The combination index (CI) was determined by titrating HSV1716-GFP (MOI = 0, 0.01, 0.1, 1, 10) with alisertib (0, 15, 31, 62, 125, 250, 500, 1000 nM) and synergy was calculated using the Chou-Talalay method of synergy analysis with the Compusyn Software and then CI value plotted versus the fraction affected. For the *in vitro* assays, all data points represent mean ± SD, (*n* = 4 measurements per well). All the experiments were repeated 3 times. Representative data are shown.

To study the mechanism underlying the enhanced efficacy in the alisertib plus HSV1716 cohorts, we first determined if alisertib and HSV1716 act synergistically to kill cancer cells *in vitro*. We exposed S462TY and SK-N-AS cells to various doses of HSV1716-GFP with or without alisertib. While each agent alone slowed cell growth, the two in combination prevented S462TY and SK-N-AS cell growth altogether (Figure 3A and 3B respectively). In addition, the two agents were synergistic at nearly all concentrations tested as determined by the Chou-Talalay combination index (Figure 3C and 3D). Typically, combination indices less than 0.9 indicate synergy, 0.9-1.1 indicate additivity, and greater than 1.1 indicate antagonism.

**Figure 4:**
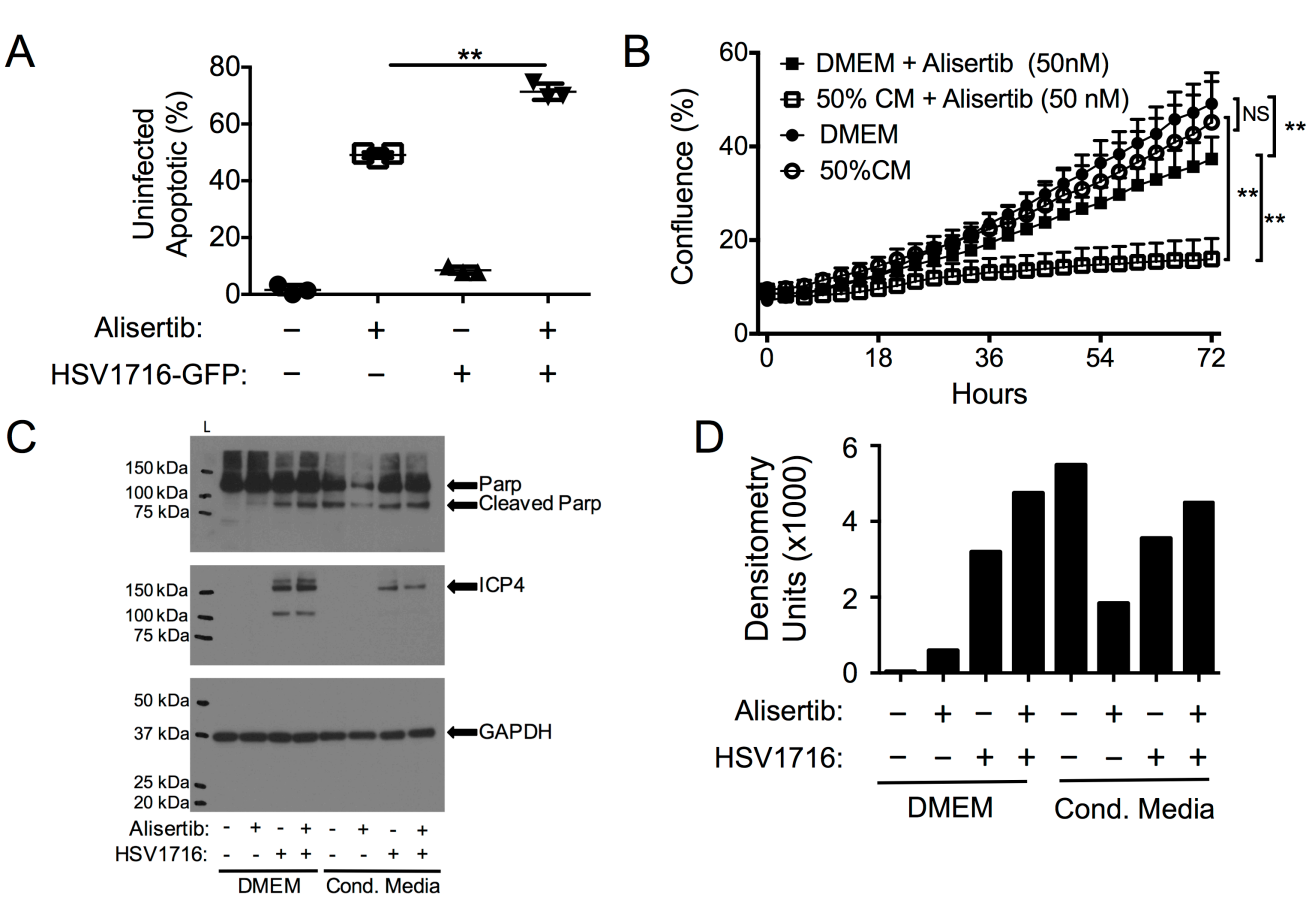
Virus-Induced Therapeutic Adjuvant (VITA) sensitizes target cells to alisertib therapy **A**. Apoptosis (Annexin V positive by flow cytometry) in uninfected S462TY cells identified by GFP negativity in cultures exposed to HSV1716-GFP and/or alisertib. **B**. Effect of 50% conditioned media (CM) from HSV-infected cells on S462TY sensitivity to alisertib. **C**. Western blots of cleaved PARP and virus ICP4 expression in S462TY cell lysates exposed to alisertib and/or HSV1716 when grown in DMEM or Conditioned Media. **D**. Densitometry of cleaved PARP for each sample in the blot shown in (C). **P < 0.01 as determined by 2-way ANOVA.

Previous reports have suggested that cells infected with HSV-1 can release byproducts that kill uninfected neighboring cells [22, 23]. We wondered if such a paracrine signal might cause uninfected cells to be more sensitive to alisertib cytotoxicity, contributing to the synergy. To test this hypothesis, we infected cells with HSV1716-GFP and measured apoptosis (annexin-V staining) in uninfected (GFP-negative) cells. In both cell lines, virus infection caused a small increase in dead cells in uninfected cells (<10%), consistent with previous observations of a bystander effect (Figure 4A). Furthermore, uninfected cells showed significantly more apoptosis in the cultures treated with the combination than in those treated with alisertib alone. To determine if infected cells produced a bystander effect, we also examined the effects of cultured media derived from virus-infected cells that had been filtered to eliminate residual viral particles. This conditioned media, which was supplemented 50% with normal media to ensure the presence of sufficient nutrients, was itself cytotoxic to MPNST and neuroblastoma cells and increased alisertib cytotoxicity. We found the conditioned media from human cancer cells was less potent than that from mouse 3T6 fibroblasts (not shown), which were used for subsequent experiments (Figure 4B). The cytotoxicity due to conditioned media was due at least in part to increased apoptosis as determined by PARP cleavage, which increased compared to those observed in normal media when cells were incubated in conditioned media from virus-infected cells and exposed to different treatments (Figure 4C and 4D). These data are consistent with a paracrine effect whereby infected cells secrete signals that kill some uninfected cells and act as an adjuvant to alisertib-medited cytotoxicity. Therefore, we coined this paracrine effect virus-induced therapeutic adjuvant, or VITA.

### Virus pretreatment enhances synergy

**Figure 5:**
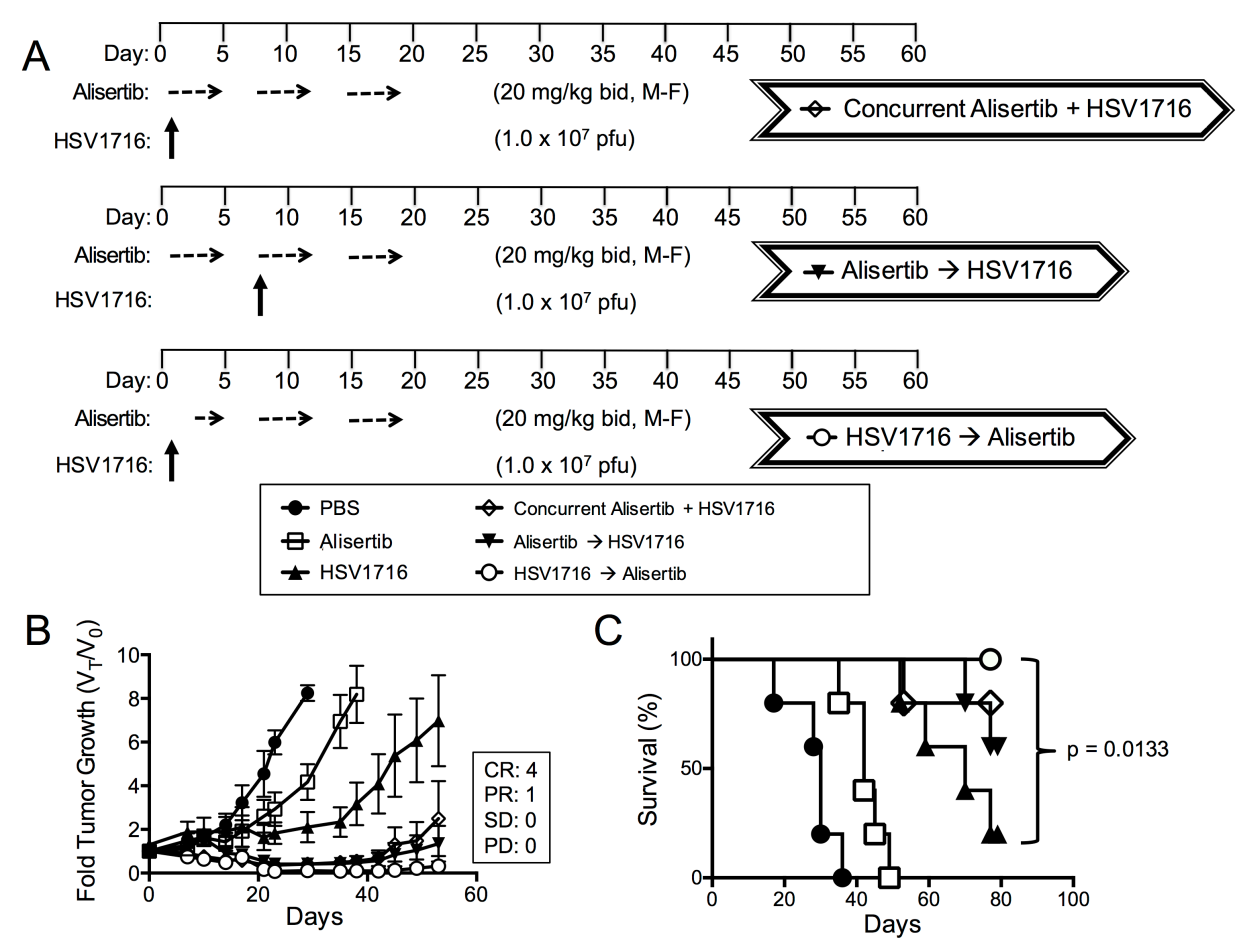
Virus pretreatment enhances synergy **A**. Dosing schema for mice bearing subcutaneous S462TY xenograft tumors. Mice were first given alisertib (20 mg/kg) or vehicle by oral gavage (twice daily 5 days per week, M-F) for 19 days in addition to an intratumoral injection of HSV1716 (1 × 10^7^ pfu) or PBS on day 1. Another cohort of mice bearing subcutaneous xenograft tumors received alisertib (20 mg/kg) or vehicle by oral gavage (twice daily 5 days per week) for 19 days in addition to an intratumoral injection of HSV1716 (1 × 10^7^ pfu) on day 5. A final cohort of mice bearing subcutaneous S462TY xenograft tumors were administered a single injection of HSV1716 (1 × 10^7^ pfu) on day 1 followed by alisertib (20 mg/kg) initiating on day 3 by oral gavage (twice daily for, 5 days per week) for a period 16 days. **B**. S462TY tumors were measured twice per week and growth plotted as fold tumor growth. Clinical responses were categorized for each tumor in the combination group as shown complete response (CR), partial response (PR), stable disease (SD) or progressive disease (PD). **C**. Survival curves of mice in panel B; mice were sacrificed when the tumor volume reached 1500mm^3^ (*n* = 5, p value for survival determined by log-rank).

Based on this *in vitro* paracrine effect, we predicted that administration of virus prior to alisertib would be more effective than administration of virus given concurrently or after alisertib. Consistent with this idea, we found the tumor response to administration of alisertib and HSV1716 concurrently was slightly diminished with 1 CR, 3 PR and 1 SD (Figure 5A) compared to the initial regimen of dosing alisertib for 5 days prior to virus, which yielded 2 CR and 3 PR (Figure 1B). Importantly, the mice treated with HSV1716 followed by alisertib reached CR in 80% of the mice (Figure 5B). We did not find a significant difference in overall survival between the combination cohorts (Figure 5C).

### Alisertib prolongs intratumoral HSV1716 viral persistence

**Figure 6:**
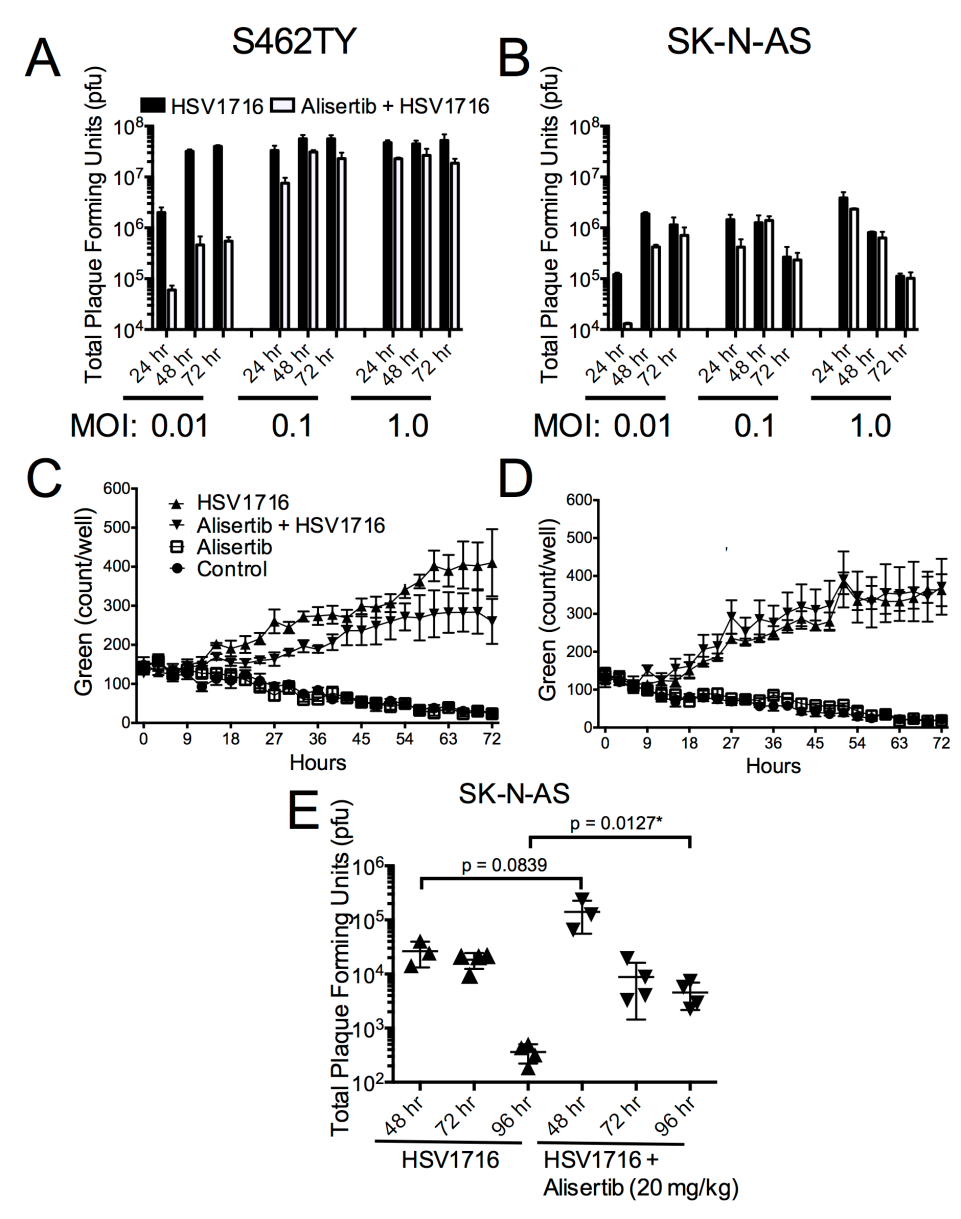
Alisertib decreases virus replication ***in vitro*** but enhances HSV1716 replication and persistence ***in vivo***. **A**. S462TY tumor cells were treated with alisertib (80 nM) or vehicle for 48 hours and then infected with HSV1716 at the various MOI indicated. Cells were harvested at 24, 48 and 72 hours post virus injection and analyzed for virus replication by plaque assay. For the *in vitro* replication assays, all data points represent mean +SD, (*n* = 4 replicates per MOI at each time point). **C**. and **D**. S462TY or SK-N-AS tumor cells were treated with alisertib (80 nM) or vehicle for 48 hours and then infected with HSV1716-GFP (MOI 0.1). Green fluorescence was monitored over time using the Incucyte. For the *in vitro* cytotoxicity assays, all data points represent mean ± SD, (*n* = 4 measurements per well). All the experiments were repeated 3 times. Representative data are shown. **E**. Mice bearing SK-N-AS xenograft tumors were treated with alisertib (20 mg/kg twice daily) or vehicle and given a single intratumoral injection of 1 × 10^7^ pfu HSV1716, and the amount of infectious virus recovered at various times determined by plaque assay. For the *in vivo* replication studies, all data points represent mean ± SD, (*n* = 3-4 replicates per time point).

The mild synergy we observed in cell lines may not completely account for the large combination effect *in vivo*. To establish if augmented HSV1716 replication might also play a role, we measured the effect of alisertib on virus production *in vitro*. Alisertib caused a slight decrease of virus production over time in both tumor cell lines following virus infection (Figure 6A and 6B). Consistent with these findings, we observed slightly decreased virus transduction in cultured cells by measuring fluorescence from GFP-expressing HSV1716 (Figure 6C and 6D). Together these results suggest that alisertib does not enhance cell autonomous virus infection kinetics.

Our findings were somewhat different using tumors grown *in vivo* (Figure 6E). Interestingly, the HSV1716 titer in the alisertib treated tumors peaked slightly higher at 48 hours, and the amount of recoverable infectious virus was 10-fold above the control tumors at 96 hours. These data suggest that alisertib increases levels and persistence of HSV1716 *in vivo*.

### Alisertib prevents virus-mediated increase in intratumoral innate immune cells

**Figure 7:**
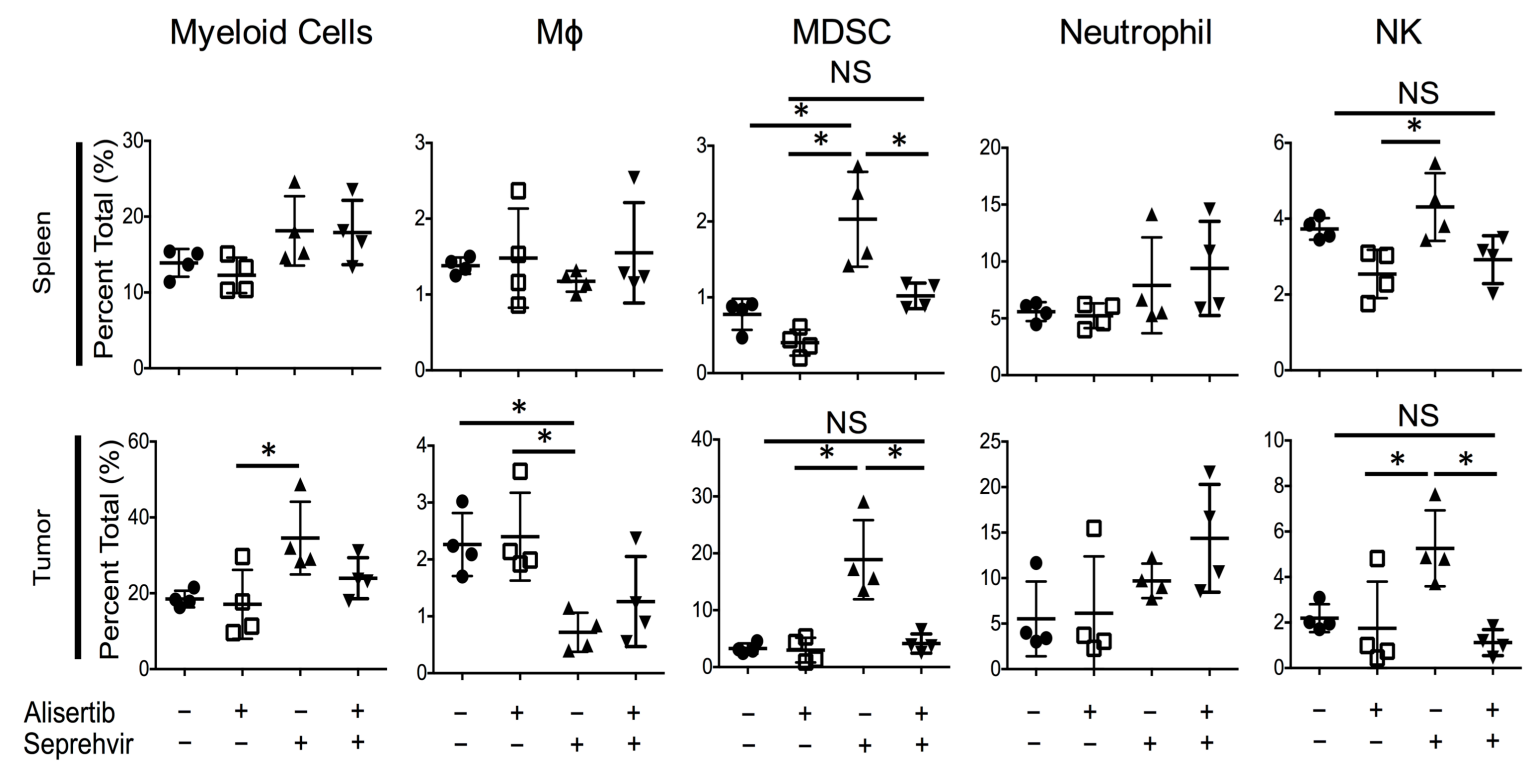
Alisertib changes virus-induced immune cellular composition Mice bearing S462TY xenograft tumors were treated with alisertib (20 mg/kg twice daily) or vehicle and given a single intratumoral injection of 1 × 10^7^ pfu HSV1716. Tumors and spleen were harvested 3 days later and immune cell compositions were quantified by single cell flow cytometry. Myeloid Cells: CD11b^+^ cells in live gate. Macrophages: CD11b^+^F4/80^+^ cells in live gate. mMDSC: CD11b^+^Ly6c^hi^Ly6g^lo^ cells in live gate. Neutrophils: CD11b^+^Ly6c^lo^Ly6g^hi^ cells in live gate. NK cells: F4/80^-^CD49B^+^ cells in live gate. For the *in vivo* cellular infiltrate studies, all data points represent mean ±SD (*n* = 4 replicates). Experiments were repeated 2 times.

We previously observed in xenograft models of neuroblastoma that oncolytic HSV induces an intratumoral infiltration of innate inflammatory cells (unpublished). Because alisertib increased HSV1716 kinetics *in vivo* but not *in vitro*, we postulated that alisertib might alter the tumor immune microenvironment. In S462TY tumors we found that virus alone increased intratumoral Ly6c^hi^ monocytic myeloid derived suppressor cells (mMDSC), natural killer (NK) cells, and Ly6g^hi^ tumor associated neutrophils (gMDSC) (Figure 7). Interestingly, alisertib prevented the increase in mMDSCs and NK cells without affecting virus-induced changes in neutrophils or macrophages. Fewer NK cells following virus infection as a result of alisertib treatment may explain the higher and more persistent virus titers we found in tumors.

## DISCUSSION

Alisertib is a viable clinical therapeutic shown to be safe in single agent and combination studies. Additionally, alisertib is an effective clinical therapeutic shown to induce stable disease and partial responses in phase I and II studies [24, 25]. Alisertib is of particular interest for neuroblastoma due to preclinical efficacy in xenograft models [21] and the discovery that Aurora A Kinase stabilizes the oncogene MYCN [26, 27]. Phase I studies in children showed that alisertib is safe both as a single agent and in combination with chemotherapy. Some patients in these early phase trials experienced stable disease and partial responses across several different tumor types [28, 29]. As a single agent, alisertib also produced prolonged stable disease in some patients with MPNST [30].

Talimogene Laherparepvec (T-VEC) is a modified herpes virus that recently was given FDA approval for melanoma by intralesional injection based on favorable phase III clinical trial data [31]. HSV1716, like T-VEC, is attenuated by deletion of the ICP34.5 genes, and shows widespread cytolytic activity against a variety of cancer types, including neuroblastoma [12]. Other herpes virus mutants have also been shown to have activity in preclinical models of neuroblastoma [11, 32] and MPNST [13, 14, 33–35]. HSV1716 demonstrated safety in several clinical trials after delivery by intracranial and intralesional injections [16–19]. A phase I safety trial in children and young adults is also currently underway (www.clinicaltrials.gov NCT00931931). As with most cancer therapies, however, combination therapies may be required to fully leverage the anti-tumoral efficacy of oncolytic herpes simplex viruses.

**Figure 8:**
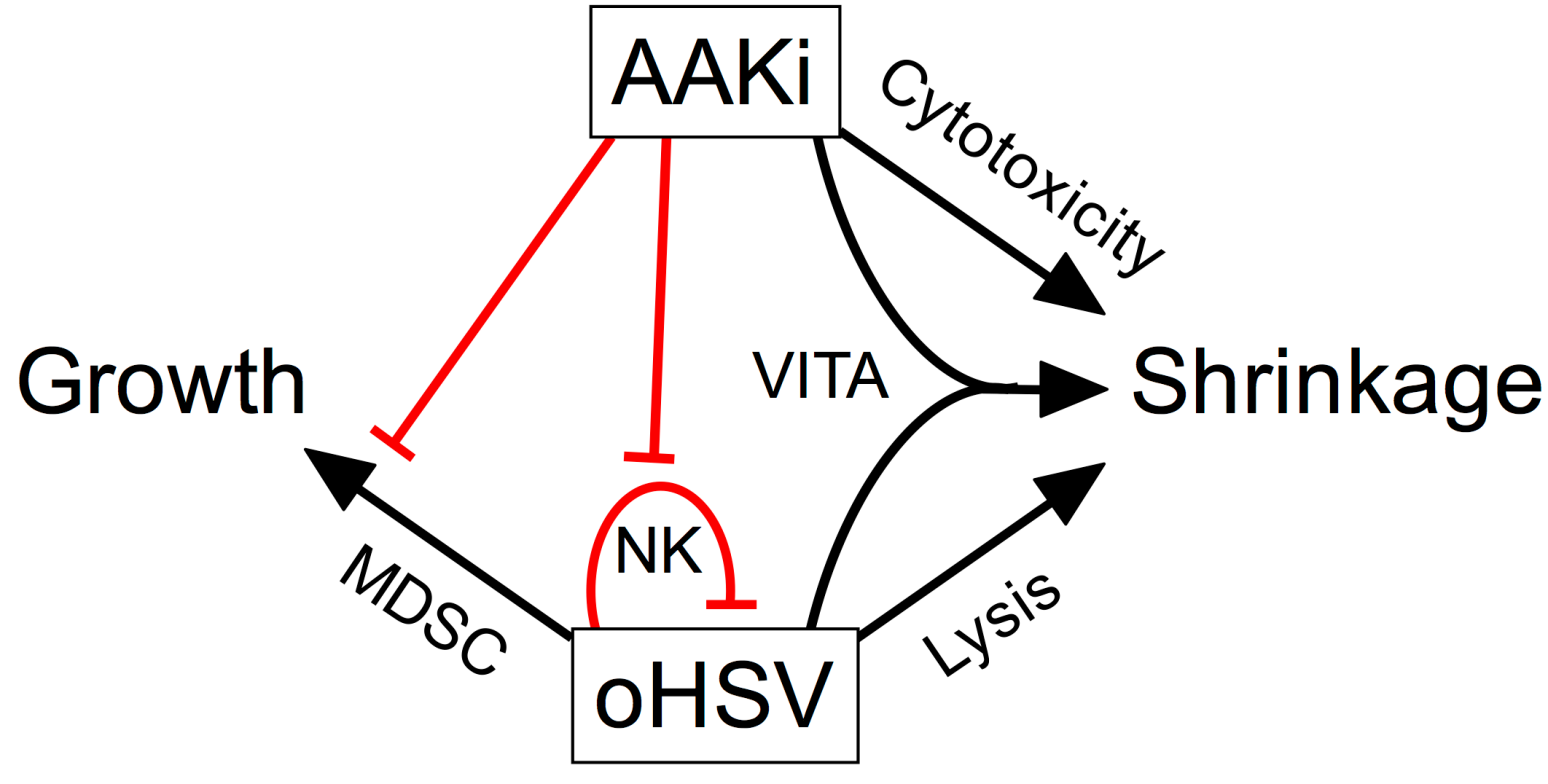
Model of pleotropic effects of alisertib on oncolytic herpes virotherapy Our data suggest a model whereby inhibition of aurora A kinase combines with virotherapy in multiple ways to elicit tumor shrinkage. Alisertib causes direct cytotoxicity and virus induces direct cell lysis. In addition, virus infection sensitizes surrounding cells to alisertib, an effect termed virus-induced therapeutic adjuvant (VITA). Virus infection also induces NK and mMDSC infiltration and/or proliferation, which is prevented by alisertib. The loss of NK cells slows virus clearance, enabling more prolonged oncolysis. The prevention of mMDSCs may also play a role as these cells can differentiate into M2-like macrophages and promote tumor growth.

We found that inhibition of Aurora A Kinase activity with alisertib used in combination with oncolytic herpes virus improves antitumor efficacy in neurogenic tumor models. The combination effect was due in part to synergistic cytotoxicity. Synergy appears to result from a bystander effect that renders uninfected cells more susceptible to alisertib. *In vivo*, alisertib slows virus clearance from tumors, likely due to mitigation of virus-induced NK cells that would normally be expected to kill virus-infected cells, prolonging oncolysis and contributing to antitumor efficacy. Indeed, the NK cell response was shown to diminish virolytic effects in preclinical models of brain tumors [36]. In addition, alisertib had other effects on the tumor immune microenvironment, including the prevention of virus-induced mMDSC accumulation. The effect is likely due to a systemic inhibition of proliferation of these cells because we also observed inhibition of virus-induced mMDSC and NK cells in spleens. The prevention by alisertib of an increase in mMDSC that normally accompanies virus infection may also improve cancer virotherapy, as mMDSC are known to convert to pro-tumorigenic M2-like macrophages [37]. Our multi-mechanistic model for the effect of combination alisertib with herpes virotherapy is illustrated in Figure 8.

The mechanisms by which oncolytic viruses induce tumor shrinkage extend beyond lytic infection to include an adaptive immunotherapeutic component [15]. Virus infection can increase recruitment and activation of antitumor T cells, broaden the tumor antigens targeted by T cells [38], and synergize with T cell checkpoint inhibitors [39]. Because Aurora A Kinase is important in T cell proliferation and its inhibition impairs T cell expansion [40], one potential downside may be that alisertib could impair the immunotherapeutic effects of HSV1716, which we would not have detected in our studies using immunodeficient mice. In that regard, however, it is interesting to note that the secretion of CCL5 induced by alisertib (so-called senescence-associated secretory phenotype) in other cancer models actually increased rather than decreased tumor infiltrating lymphocytes [41]. Because most animal cells are not highly permissive to human herpes simplex virus infection, the full impact of alisertib on the integrated effects of both lytic and immunotherapeutic phases will only truly be testable through human trials.

The effect of alisertib on virotherapy may not be limited to herpes virus, as the combination of alisertib also improves the antitumor efficacy of oncolytic measles virus in several cancer models [42]. In those studies, increased virus gene expression was suggested to be a possible mechanism of interaction. Alisertib also induced expression of cellular genes, such as IL-24, which they postulated might increase antitumor effects.

Two reports using HSV-1 derivatives are consistent with our observation of a bystander effect. Stanziale and colleagues noted that virus infection increased apoptosis in uninfected cells and limited virus spread, which was reversed with an inhibitor of apoptosis [22]. Takasu et al. noted oHSV infection induced a so-called immunogenic cell death with secretion of damage associated molecular patterns ATP and HMGB1, and that intratumoral injection of supernatants from infected cells slowed tumor growth [23]. Although the exact nature of molecules that mediate the bystander effect are not known, it is possible this phenomenon will sensitize cells not only to alisertib but also to other targeted or cytotoxic drugs. Whether the bystander effect occurs with other viruses is unknown, but there are reports of other viruses synergizing with chemotherapy or targeted small molecules [43, 44].

Alisertib did not increase oHSV permissivity *in vitro*. In fact, under most conditions the combination of alisertib with oHSV reduced virus production. We attribute this finding to drug-induced senescence and cell death resulting in fewer cells available for infection. It is also possible that alisertib actually impairs virus production at the cellular level. In this regard, it is interesting to note that aurora A kinase regulates NuMA phosphorylation [45]. NuMA is a nuclear matrix protein whose cellular localization is determined by phosphorylation status and is essential for HSV assembly and egress [46]. In contrast, several other small molecules including the proteasome inhibitor bortezomib [47], doxorubicin [48], a copper chelator [49], and HDAC inhibitors [50, 51] synergize with oHSV by increasing herpes virus production. Thus multiple mechanisms exist by which co-administered drugs may enhance oncolytic herpes virotherapy.

In summary, we found that aurora A kinase inhibition by alisertib synergizes with oncolytic herpes virotherapy, likely through multiple mechanisms. Our results support testing of this combination of agents, particularly in children and young adults with neuroblastoma and MPNST.

## MATERIALS AND METHODS

### Compounds and reagents

Alisertib (a.k.a. MLN8237) was generously provided by Millennium Pharmaceuticals, a wholly owned subsidiary of Takeda Pharmaceutical Company Limited (Boston, MA). For *in vitro* studies, alisertib was dissolved in dimethyl sulfoxide (DMSO, Sigma Aldrich, St. Louis, MO) at 50 mM and then diluted to the appropriate concentrations in complete cell culture media. DMSO alone was diluted in complete cell culture media as a control for the *in vitro* assays. For *in vivo* studies, alisertib was formulated daily at 2.5 or 5.0 mg/mL in 3% ethanol / 9.7% 2-hydroxypopyl-b-cyclodextrin / 0.97% sodium bicarbonate. The monoclonal antibodies anti-CD49b-PE (Clone DX5), anti-F4/80-PECy7 (Clone BM8), anti-Ly6G-APC-Cy7 (Clone 1A8) and anti-CD11b-Violet 421 (Clone M1/70) were purchased from Biolegend (San Diego, CA). Monoclonal antibody anti-Ly6C-APC (AL-21) was procured from BD Biosciences (San Jose, CA). Anti- poly-ADP-ribose polymerase (11835238001) antibody was purchased from Roche Indianapolis, IN). Anti-ICP4 (Clone 10F1) antibody was obtained from Virusys Corporation (Taneytown, MD), and anti-GAPDH (Clone 14C10) antibody was obtained from Cell Signaling Technology (Danvers, MA).

### Viruses

HSV1716 (HSV1716) and HSV1716-GFP, an engineered mutant that expresses green fluorescent protein under the control of the CMV promoter, were generously provided byVirttu Biologics (Glasgow, U.K.). HSV1716 is derived from the HSV-1 strain 17+, but has been engineered with a deletion of the gene encoding ICP34.5, reducing its virulence and causing it to be permissive for replication selectively in rapidly dividing cells [52, 53]. The non-replicating virus control, UV-HSV1716, was prepared by exposing a vial of HSV1716 to a germicidal ultraviolet light. Briefly, 1.5 mL of HSV1716 (5 x10^8^ pfu/mL) was placed in an open petri dish on ice in a Nuaire Labgard Class II, Type A2 Laminar Flow Biological Safety Cabinet and directly exposed to the germicidal ultraviolet light at 253.7 manometers with an average intensity of 100 microwatts per centimeter for 45 minutes. The UV-inactivated HSV1716 sample was then confirmed by standard viral plaque assay to be negative for plaque forming units (pfu) prior to experimental use.

### Cell lines and cultures

The history and cell culture maintenance of tumor cell lines S462TY, a human malignant peripheral nerve sheath tumor, and SK-N-AS, a human neuroblastoma, were previously reported [12, 33]. Briefly, S462TY and SK-N-AS cells were grown in Dulbecco's modified essential media (DMEM) supplemented with 10% fetal bovine serum (FBS) and 1% penicillin/streptomycin. In addition, all cells were cultured at 37°C in a humidified incubator at 5% CO_2_. Both cell lines were subjected to routine short tandem repeat genotyping analysis (Molecular Genetics Core, Nationwide Children's Hospital, Columbus, OH) and mycoplasma testing using the MycoAlert™ Mycoplasma Detection Kit (LT07-318, Lonza Inc. Allendale, NJ).

### Preparation of conditioned media

3T6 mouse fibroblasts were used to produce HSV1716-conditioned media. Briefly, 3T6 cells were allowed to reach 50% confluence, washed one time with phosphate buffered saline (PBS), and fresh DMEM (10% FBS, 1% penicillin/streptomycin) was added. 3T6 cells were then infected by a MOI of three with HSV1716, UV-inactivated HSV1716, or PBS and incubated for 24h at 37°C 5% CO2 under sterile conditions. Supernatants were collected and centrifuged at 4,000 g for 10 minutes. Supernatants were then filtered using the SteriFlip vacuum-driven filtration system (0.22 μm, EMD Millipore, Billerica, MA). Finally, supernatants were then UV-irradiated for 45 minutes on ice to inactivate any remaining competent virus. All supernatants were confirmed to be virus-free by standard plaque forming assay.

### Immunohistochemistry

Formalin fixed tissues were paraffin embedded, sectioned at 5 μM thickness, baked at 60°C for 1 hour and air dried. We deparaffinized, hydrated, transferred sections to 0.1M citrate buffer (pH 6.0) for antigen retrieval, boiled for 10 minutes, then cooled at room temperature for 30 minutes. Sections were quenched with 3% hydrogen peroxide for 10 minutes, rinsed in PBS, and blocked in normal goat serum (10% serum with 0.3% Triton-X-100 in PBS) for 1 hour. Then, sections were incubated overnight in primary antibody diluted in blocking solution. We used rabbit anti-Ki67 (Novocastra; 1:5,000). After PBS rinses, sections were incubated in biotinylated goat anti-rabbit secondary antibodies (Abcam, Cambridge, MA) for 1 hour at room temperature, rinsed in PBS, incubated in ABC reagent (Vector Laboratories, Burlingame, CA) for one hour, rinsed in PBS and incubated in DAB (Vector Laboratories) for 5 minutes. After DAB, sections were rinsed, dehydrated in series of graded alcohols (70%, 95%, and 100%), cleared in Xylenes and finally cover glassed in Histomount (Life Technolgies, Carlsbad, CA). We acquired microscopic images with Openlab software suites on a Zeiss Axiovert 200.

### *In vitro* synergy assays

To determine two-drug synergy *in vitro*, cells were seeded at 2,000 cells per well in a 96 flat bottom well plate and allowed to attach overnight. Cells were then washed with sterile PBS. Fresh media was added containing HSV1716-GFP (MOI = 0, 0.01, 0.1, 1, 10) alone, alisertib (0, 15, 31, 62, 125, 250, 500, 1000 nM) alone or the combination of the HSV176-GFP and alisertib at the various MOI and concentrations, respectively. Cell proliferation was monitored by phase contrast imaging every 3 hours using the IncuCyte Zoom time lapse imaging system (Essen Biosciences, Ann Arbor, MI, USA). Synergy was determined using the Chou-Talalay method of synergy analysis [54] using CompuSyn for Drug Combinations Analysis Software (ComboSyn, Inc., Paramus, NJ, USA).

### *In vitro* virus replication

Tumor cells were seeded in 12-well tissue culture dishes at a concentration of 1.75×10^5^ cells per well and incubated overnight at 37°C in a humidified incubator at 5% CO_2_. After replacing the culture media with fresh media containing alisertib at 0 or 80 nM, the cells were then incubated for an additional 48 hours. Prior to infecting the cells with virus, representative control and alisertib treated cells were counted by trypan blue exclusion to determine the appropriate virus infection concentration. After washing cells and adding fresh media containing alisertib at 0 or 80 nM, cells were infected with HSV1716 at a multiplicity of infection (MOI) = 0.01, 0.1 and 1.0. Infected cells were harvested at 24, 48 and 72 hours post virus infection by cell scraping. Harvested cells and media were freeze-thawed three times and then assayed for total plaque forming units via standard plaque assay.

### Cell permissivity assay

To monitor virus infection and spread, S462TY or SK-N-AS cells were seeded at 2,000 cells per well in a 96 flat bottom well plate and allowed to attach overnight. Cells were washed with sterile PBS, then either fresh media or conditioned media (prepared from virus-infected cells, see above) was then added to each well and treated with or without HSV1716-GFP (MOI = 0.5) in the presence or absence of alisertib (50 nM). Virus infection was monitored by phase contrast and GFP-imaging every three hours using the IncuCyte Zoom time lapse imaging system (Essen Biosciences, Ann Arbor, MI, USA).

### Animal studies

All animal studies were approved by the Institutional Animal Care and Use Committee (IACUC) for the Research Institute at Nationwide Children's Hospital. Female athymic nude mice, age 4-6 weeks and weighing 15-25 g were purchased from Envigo (Indianapolis, IN). These mice were subcutaneously injected with 5.0 x10^6^ S462TY or 1.0 x10^7^ SK-N-AS cells in 150 μL mix of phosphate-buffered saline (PBS) and matrigel (2:1). Alisertib was formulated at 2.5 or 5 mg/mL in the vehicle described above. Alisertib or vehicle was administered by oral gavage in 100 μL. HSV1716 was diluted in PBS to 1.0 x10^8^ pfu/mL and administered intratumorally in a final volume of 100 μL. PBS was administered intratumorally as the control for HSV1716 in a final volume of 100 μL.

### Efficacy studies

When the tumors reached a mean volume between 175 - 250 mm^3^, the mice were randomized into four study groups: vehicle + PBS, alisertib + PBS, vehicle + HSV1716 and alisertib + HSV1716. The alisertib dosing schema and dose was varied to determine the most efficacious regimen and if a clinically relevant dose could be achieved. UV- HSV1716 was used in a control to determine if virus replication was essential. The specific dosing schema is described for each experiment in the figure legends. Animals were followed until the animal reached endpoint criteria, including tumor volume > 1500 mm^3^, body weight loss > 20%, unusual mouse behavior, lack of movement or poor posture. Tumor size was measured using calipers twice per week and tumor volume was calculated using the following formula: a x b2(π/6) where a is the longest diameter and b is the shortest diameter. Mice were also weighed and observed 2 times per week for signs of endpoint condition. Mice that demonstrated signs of toxicity or reached endpoint criteria were humanely euthanized by CO_2_ asphyxiation and subjected to cervical dislocation as the secondary method of euthanasia.

### Virus replication studies

When the tumors reached a mean volume >400 mm^3^, the mice were randomized into four study groups: PBS + vehicle, PBS + alisertib, HSV1716 + vehicle and HSV1716 + alisertib. A single dose of HSV1716 was injected intratumorally on day 0 followed by oral gavage administration of vehicle or alisertib (20 mg/kg twice daily) at 24, 48 and 72 hours post infection if the tumor was not being harvested on that day. Tumors were harvested at 48, 72 and 96 hours post infection and then assayed for virus titer using the standard plaque assay as previous described [55].

### Cell infiltrate studies

When the tumors reached a mean volume >400 mm^3^, the mice were randomized into four study groups: PBS + vehicle, PBS + alisertib, HSV1716 + vehicle and HSV1716 + alisertib. A single dose of PBS or HSV1716 was injected intratumorally on day 0 followed by oral gavage administration of vehicle or alisertib at 24 hours and 48 hours post infection. Tumors and spleens were harvested at 72 hours post infection and then prepared for flow cytometric analysis. Briefly, fresh whole spleens were transferred to 2 mL PBS and then smashed through a 70 μM cell strainer with 5 mL syringe plunger. Whole tumors were transferred to 6-well dishes containing 2 mL PBS and then processed as previously described [56]. Briefly, tumors were minced by mechanical chopping and incubated in PBS containing 25 μg/mL liberase blendzyme (Roche Diagnostics., Indianapolis, IN) and 150 μg/mL DNAse I for 1 hour at 37°C. Tumor samples were then passed through a 70 μM cell strainer with the aid of a 5 mL syringe plunger. After washing splenocytes and tumor cell suspensions once with PBS, the cell suspensions were cleared of red blood cells (RBCs) with ACK lysing buffer (Lonza, Walkersville, MD). The cells were then blocked with 5% mouse Fc blocking reagent (Miltenyi Biotec Inc., San Diego, CA) in FACS buffer (1% FBS and 1mM EDTA in PBS) and analyzed by flow cytometry.

### Western blot

S462TY or SK-N-AS cell cultures in 6-well dishes were treated with HSV1716 (MOI = 0.5), alisertib (50 nM) or both for 24 hr. Cells were washed then lysed with Cell Lysis Buffer (Cell Signaling Technology, Danvers, MA) supplemented with Halt Protease Inhibitor Cocktail (Thermo Fisher Scientific, Waltham, MA). Cell lysates were quantified then heat denatured using 10μg protein loaded per lane in a 4-12% gradient bis-tris gel (Invitrogen, Carlsbad, CA). Protein was transferred to a nitrocellulose membrane and blotted with antibodies against poly-ADP-ribose polymerase (Roche, Indianapolis, IN), ICP4 (Virusys, Taneytown, MD), and GAPDH (Cell Signaling Technology, Danvers, MA). Densitometry analysis was performed using ImageJ software (NIH, Bethesda, MD).

### Quantitation of apoptotic cells

Sub-confluent S462TY or SK-N-AS cell cultures in 6-well dishes were treated with HSV1716-GFP (MOI = 0.5), alisertib (50 nM) or both for 72 hours. Cells were then harvested, washed twice with cold PBS, stained using the Annexin (APC)/Propidium Iodide (PI) kit according to the manufacturer's instructions (BioLegend, San Diego, CA), and analyzed by flow cytometry for the presence of apoptotic cells. Apoptotic cells were gated on annexin positive, HSV1716-GFP-negative cells to include all early and late apoptotic HSV-negative cells.

### Statistics

Plot generation and data analysis was performed using GraphPad Prism Version 6.0 (La Jolla, CA). Statistical significance amongst groups for *in vitro* assays and cell infiltration studies was determined by two-way analysis of variance (ANOVA). Log-rank test was used to determine significance for survival studies. Significance is reported as * (*P* < 0.05), ** (*P* < 0.01). *P* values <0.05 were considered significant. *In vivo* synergy was determined using the Bliss Independence Model [57].

### Flow cytometry

For cellular infiltrate studies, 1 × 10^6^ cells were stained with antibodies against CD49b, F4/80, Ly6G, CD11b and Ly6C according to standard surface staining protocol. Stained cells were then fixed in 0.5% paraformaldehyde and data acquired on a BD FASC LSR II (BD, Franklin Lakes, NJ). For apoptotic cell quantitation, cells were processed as described in “quantitation of apoptotic cells” section and then data acquired on a BD FASC LSR II. Further analysis for both studies was carried out using the FlowJo software, version 10.0.3 (Tree Star Inc., Ashland, OR).

## Abbreviations

ANOVA: Analysis of variance
CD11b: cluster of differentiation 11b
CD49b: cluster of differentiation 49b
CR: Complete response
DAB: 3,3’-diaminobenzidine
DMEM: Dulbecco’s modified essential media
DNase: deoxyribonuclease
EIF2α: Eukaryotic translation initiation factor 2, subunit 1 alpha
EDTA: Ethylenediaminetetraacetic acid
FBS: Fetal Bovine Serum
GAPDH: Glyceraldehyde 3-phosphate dehydrogenase
GFP: Green Fluorescent Protein
gMDSCs: Granulocytic myeloid derived suppressor cells
HDAC: Histone deacetylase
HMGB1: High mobility group box 1 protein
HSV1716: Herpes Simplex Virus 1716
ICP4: Infected Cell Protein 4
ICP34.5: Infected Cell Protein 34.5
Ly6c: lymphocyte antigen 6 complex, locus C1
Ly6g: lymphocyte antigen 6 complex, locus G
MAP kinase: Mitogen-activated protein kinase
mMDSCs: Monocytic myeloid derived suppressor cells
MOI: Multiplicity of Infection
MPNST: Malignant Peripheral Nerve Sheath Tumor
MYCN: v-myc avian myelocytomatosis viral oncogene
NK: Natural Killer
NuMA: nuclear mitotic apparatus protein 1
oHSV: Oncolytic Herpes Simplex Virus
PARP: Poly ADP ribose polymerase
PBS: Phosphate Buffered Saline
PD: Progressive disease
PFU: Plaque forming unit
PKR: Protein kinase R
PR: Partial response
RBC: Red blood cell
SD: Stable disease
UV: Ultraviolet
VITA: virus-induced therapeutic adjuvant
Data collection, editing: AVP.
Writing, editing: JAE, JC.
Data collection: MRF, KEC.
